# Purpura with regular shape in an adolescent: Beware of dermatitis artefacta

**DOI:** 10.3389/fped.2022.959064

**Published:** 2022-11-03

**Authors:** Yuhao Chen, Lin Li, Jing Lu

**Affiliations:** ^1^ Department of Pediatrics, West China Second Hospital of Sichuan University, Sichuan, China; ^2^ Key Laboratory of Birth Defects and Related Diseases of Women and Children, Sichuan University, Ministry of Education, Sichuan, China; ^3^ Pathology Department, West China Hospital of Sichuan University, Sichuan, China

**Keywords:** dermatitis artefacta, painful purpura, rapid diagnostic, psychiatric disease, multidisciplinary team

## Abstract

**Background:**

Purpura is common in pediatric patients, mostly diagnosed as IgA-related vasculitis (Henoch–Schönlein purpura), idiopathic thrombocytopenic purpura (ITP), and thrombotic thrombocytopenic purpura (TTP). However, in some cases, for example, cases with dermatitis artefacta, it could puzzle a physician or pediatrician for a long time, with great challenges in diagnosis.

**Case presentation:**

We present the case of a 13-year-old boy with recurrent painful purpura on both upper limbs. The physical exam was unremarkable, except for right blepharoptosis and scars from burns. The diagnostic tests were normal. Through repeated communication, the patient was finally diagnosed as having dermatitis artefacta, accompanied by underlying psychological problems.

**Conclusions:**

Before dermatitis artefacta was diagnosed, we spent a lot of money and effort on the diagnosis. Therefore, in order to determine the diagnosis as soon as possible and save on unnecessary medical expenses, we propose a rapid process for the diagnosis of purpura of dermatitis artefacta in children.

## Introduction

Purpura is a common manifestation in pediatric patients mostly diagnosed as IgA-related vasculitis (Henoch–Schönlein purpura [HSP]) (1), systemic lupus erythematosus (SLE), idiopathic thrombocytopenic purpura (ITP), thrombotic thrombocytopenic purpura (TTP) (2), and so on. However, purpura with a regular shape or bizarre distribution may puzzle a physician for some time (3, 4). After the exclusion of organic diseases, a diagnosis of dermatitis artefacta (DA) can be carefully considered. DA is a relatively rare condition in children, which presents an array of different types of skin manifestations, including superficial erosions, excoriations, and purpura (5, 6). Often, the presence of purpura in children attracts particular concern from physicians, as there may be potentially serious pathological consequences that require intervention as soon as possible. However, information on DA in children and adolescents is still limited.

The skin is the largest organ in the human body, which lines the outer surface. From an embryological point of view, both the skin and nervous system are derived from the ectoderm (7). Therefore, in clinical medicine, diseases of the skin and nervous system are sometimes found to be closely related (8, 9). Psychodermatology (10, 11) is a relatively new field of medicine. Even though nowadays pediatric psychodermatology attracts increasing attention from scholars, there is still limited literature available. DA, also called factitial dermatitis, is a condition caused by self-induced skin damage by an individual with an underlying emotional purpose, psychiatric disorder, or external stressor (6, 12). Diagnosing the disease is difficult because patients often do not admit that the skin rash was caused by themselves or simply made unconsciously. To our knowledge, this is the first article to focus on DA in children and propose a rapid diagnostic process.

## Case presentation

**Figure 1:**
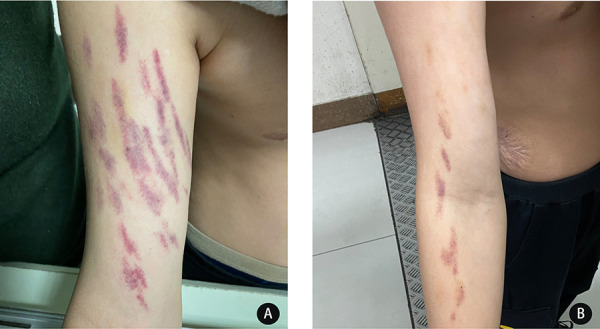
Striking linear symmetric purpuric streaks on the proximal extensor sides of both arms. The purpura was not palpable. (**A)** Purpura formed suddenly, (**B)** purpura 2 days after episode.

A 13-year-old boy was admitted to the West China Second University Hospital with the chief complaint of recurrent painful skin lesions on both upper limbs, which had lasted more than 2 years before his referral (since he was 11 years old). The boy had recurrent painful purpura on both arms, but there seemed to be no relationship between the pain site and the ecchymosis site. The purpura often flared up suddenly at night or after going to the bathroom, where he was alone. A physical examination revealed remarkable linear, symmetric (meteor shower-like) purpura on both arms (Figure 1). He had once been diagnosed with an allergic rash, or Schönlein purpura, and given glucocorticoid and other anti-allergic treatment. Although the ecchymosis subsided, this type of skin lesion was easy to relapse. In the latest episode, ecchymosis on both upper limbs reappeared, with the same morphology and distribution, and with pain all over the body. He soon complained of blindness in his right eye, but his vision gradually recovered half a month later, after treatment with eye drops (timolol maleate and sodium hyaluronate). It is interesting to note that whether he was given naproxen or only placebo, his pain was quickly relieved. The patient’s history revealed that he was born prematurely, at a gestational age of approximately 32 weeks. He had congenital right blepharoptosis and once received an “umbilical hernia” operation in his infancy. Later, he was admitted to our hospital for scalding, which left him with unsightly scars over the trunk. His parents and 21-year-old sister are healthy and denied a positive family history. His father had worked abroad for many years and his mother was rarely present because of her work. Another point worth noting is that he showed maturity beyond his age while communicating.

**Figure 2:**
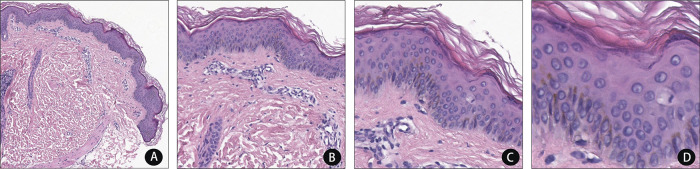
H&E stain revealed the reticular epidermis is hyperkeratosis and perivascular lymphocytes and a few neutrophils infiltrating in the dermis. (hematoxylin and eosin, original magnification A ×40, B ×100, C ×200, D ×400).

The laboratory evaluation revealed that his normal white blood cell count, erythrocyte count, platelet count, erythrocyte sedimentation rate, coagulation function, urinalysis, and hepatic and renal function were all normal. There were also no abnormal findings in humoral immunity (IgA, IgG, IgM, and IgE), cellular immunity (B cell, T cell, and NK cell), autoantibodies, antineutrophil cytoplasmic antibodies, anticardiolipin antibody, and rheumatoid factor. Chest radiographs showed a curvature of the upper thoracic scoliosis. Electroencephalogram, cerebrospinal fluid, and bone marrow examination also showed no abnormalities. A skin biopsy revealed reticular epidermis hyperkeratosis, moderate perivascular lymphocytes, and some neutrophil infiltration (Figure 2).

A multidisciplinary team was then arranged to discuss this difficult condition. After a discussion, the consultation suggested that as no clues of organic disease that could lead to purpura were found, DA caused by psychological factors should be considered. Based on the patient’s clinical history, physical examination, histopathology findings, and laboratory evaluation, the diagnosis of DA was finally made.

After discharge, we advised the patient’s parents to pay more attention to his psychological state and try their best to create a relaxing environment and provide company, which was the most important factor. During the 3-months follow-up, the patient returned to his normal life and study, without any further similar episodes.

## Discussion

### What is dermatitis artefacta?

The incidence of DA accounted for approximately 0.03% of dermatology patients, but this number is possibly underestimated (13). In fact, DA is not a disease but a phenomenon that occurs in patients who deliberately create various skin lesions: cutting, purpura, abrasion, burning, or other forms of skin lesions by means of a knife, sucking, chemical substances, scratching, and so on (14). The underlying psychiatric diseases include psychoses, intellectual disability, personality disorders, and other psychiatric pathologies, such as anxiety, depression, and posttraumatic stress disorder. In some cases, patients developed DA in order to attract attention (15). These patients often turned to a pediatrician or dermatologist first, ignoring that the underlying problem may be psychological. Saez-de-Ocariz et al. (4) suggest that the incidence of DA may be about 1 in 23,000 in pediatric patients, being mostly older children and adolescents (median age 11.17 years) (4), and girls (16–18). We search the PubMed database with the keywords “dermatitis artefacta,” and found eight articles that mentioned children with DA with purpura (Table 1). With this case, the characteristics of the artefactual purpura in children can be summarized. Interestingly, these children have similar manifestations: the distribution of DA purpura was mainly located on the upper limbs, chest, or trunk, which can be reached with both hands. The purpura often presented as linear in shape. An impressive feature is that these purpuras often occurred at night or when the child was in the bathroom, where they were alone.

**Table 1:** Comparison of published cases with artefactual purpura.

Authors	Number	Age (years)/gender	Affected aera	Distribution	Shape	Time of onset	Injury mechanism
Landers et al. (3)	2	9,14/Female	Lower back	Bilateral	U-shaped	During the bathroom	Suction
Yamada et al. (19)	1	10/Female	Flexor aspect of right upper arm, tongue, and lower thigh	Unilateral	Linear	Night	−
Gil-Bistes et al. (20)	1	12/Female	Right upper limb	Unilateral	Linear	During the bathroom	Suction
Ring et al. (21)	2	9/Male; 10/Female	Extensor aspect of arms; chin and lower lip	Bilateral	Linear; circular	Night	Suction
Hosteing et al. (22)	6	6–14/All females	Upper limbs, face, trunk, and lower limbs	Bilateral	Linear	—	Mechanically induced by self or by other
Alexander et al. (23)	1	10/Male	Arms and chest	Bilateral	Linear	—	—
Sarkar et al. (24)	3	14–18/All females	Upper limbs	Bilateral	Linear	—	Mechanically induced by themselves
Chen et al.	1	13/Male	Upper limbs	Bilateral	Linear	Night or during the bathroom	Mechanically induced by himself

### What are the manifestations and their characteristics?

#### Shape and distribution

DA can present as a variety of skin lesions but without any prodromal skin changes or descriptions of evolution. The lesions tend to be morphologically bizarre, often geometric in outline, and have a clear boundary from the adjacent normal skin (17). They even mimic organic skin diseases. Several studies concluded that superficial erosions are the most common lesions (27%), followed by purpura (17%), miscellaneous lesions (edema, panniculitis, tattoo-like, bulla, and eschar) (16%), and irritative dermatitis (11%) (4, 16, 17). Rogers et al. concluded that the common sites involved in DA included the head, neck, upper limbs, abdomen, and lower limbs (17). Lesions were usually seen within accessible areas of the patient’s hands. In our case, the boy had purpura on both arms and the skin lesions presented a regular shape, like a meteor shower in the night sky, from which point we thought this purpura might be unusual.

#### Sudden emergence of skin lesions

As the literature reported, the skin lesions of DA often appeared suddenly on previously normal skin, and sometimes occurred overnight (5). The patient in the present case report also had purpura that also suddenly occurred at night or after returning from the bathroom during hospitalization.

#### Comorbidities

Another feature is that a large proportion of patients with DA have physical diseases. In a series by Mohandas et al., a total of 10 (36%) patients (one child and nine adults) had a concomitant physical disease or a close member of the family with physical disease (5). It is possible that a long-term physical illness is psychologically stressful for the patient. In this case, excessive concern about his parents and appearance (congenital blepharoptosis) could have had a potential impact on him. In addition, the scars on his trunk undoubtedly became a stressful event for the adolescent boy, who was mostly concerned about his self-image.

#### Underlying psychophysiological disorders

It has been reported that psychosocial stress may influence a patient’s propensity to self-harm, and authors have pointed out that self-inflicting behavior, particularly if repetitive, may be a sign of underlying emotional disturbance (25). This self-inflicting behavior could be interpreted as an emotional escape valve. Therefore, it is important for us to inquire about the patient’s history in detail to understand the stressful events that he experienced. To establish trust between a physician and a patient, it has been suggested that it may be beneficial to interview the child alone, without the presence of their parents (13). In rare situations, child abuse (including Munchausen syndrome by proxy) should be taken into consideration, especially in children aged under 5 years (15).

### How to make a quick diagnosis

The bizarre morphology and geometric outline often make it particularly difficult for a physician or dermatologist to make the diagnosis. Studies by Saez-de-Ocariz et al. and Rogers et al., among others, revealed that the mean time from the onset of the disease to the time of diagnosis was 10 months, and sometimes could even take as long as 4 years (4, 17). In a study by Libow (16), the mean time from presentation to diagnosis was almost 16 months; the patients underwent numerous examinations during that time. Therefore, it is imperative that more pediatricians should be aware of the disease and be able to refer patients to the appropriate facilities for treatment.

Diagnosing DA could be a challenge because a variety of possible dermatoses can be mimicked. Although Rogers et al. argue that many examinations, including skin biopsy, are unnecessary (17), we consider that it is still necessary to exclude organic disease to diagnose this rare condition.

The clinical presentations of DA are varied, and patients rarely disclose the cause of their lesions. We focused on the presenting physical signs rather than the mode of production; factitious purpura must be differentiated from other purpura caused by organic disease, such as coagulation disorders, meningococcemia, IgA-related purpura, leukemia, polyarteritis nodosa, Ehlers–Danlos syndrome, hereditary hemorrhagic telangiectasia, psychogenic purpura (autoerythrocyte sensitization syndrome or Gardner–Diamond syndrome), and rickettsial disease (21). In addition, the recognition of vasculitis mimics is also important. For example, thrombotic and hypercoagulable conditions in patients can mimic medium and small vessel vasculitis, presenting as purpura (26, 27). In this case, the boy had no evidence of infection or history that supported simulated vasculitis. Histologically, there is no specific sign but extravasated erythrocytes and the absence of vasculitis (3).

**Figure 3:**
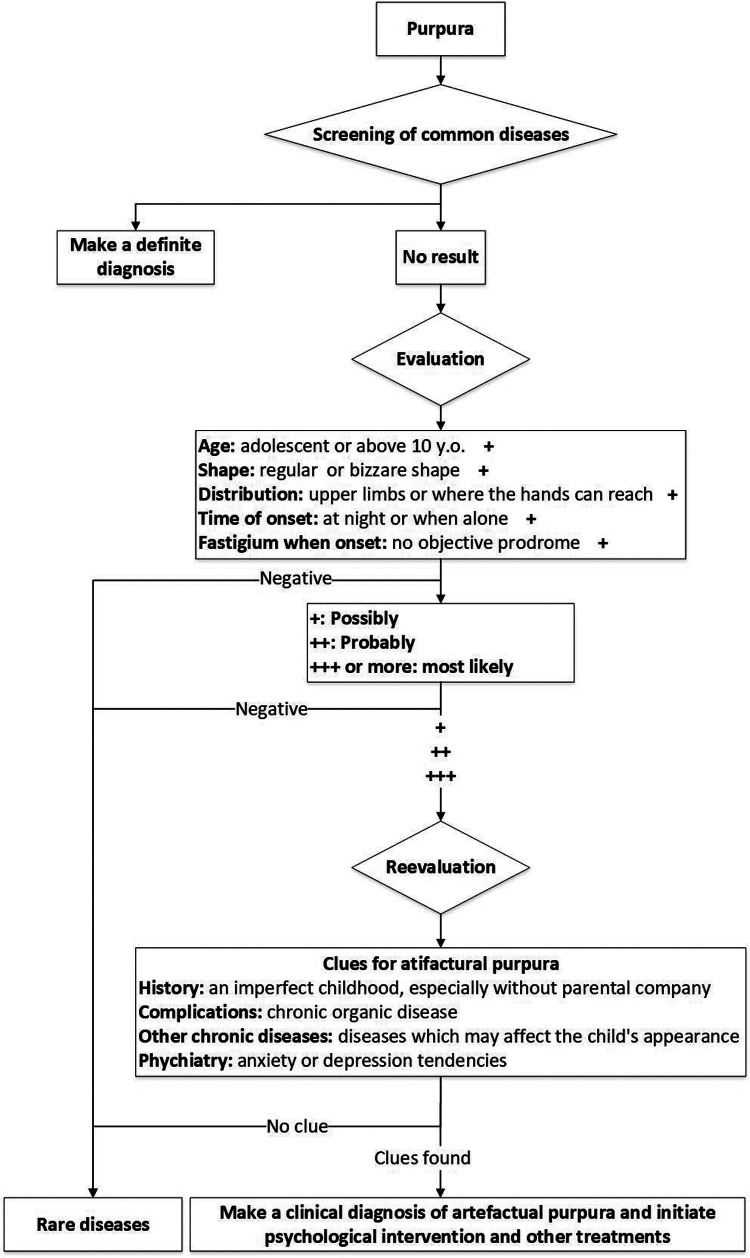
Rapid diagnostic process for artefactual purpura.

Diagnosis is made by exclusion rather than directly on the basis of histologic and biochemical findings. It is particularly common in women, adolescents, and those with an underlying psychiatric diagnosis or external stress (28). Based on the clinical disease feature, we offer a rapid diagnostic process for artefactual purpura, providing some clues (such as age above 10 years or adolescent, regular or bizarre shape of the purpura, distribution on upper limbs, and so on) for a quick diagnosis (Figure 3).

### Possible management

It is important to avoid confronting the patient and clearly explain the problem to the parents. A thorough medical history collection could reveal that the child has a family history or someone with a similar illness. If there is a delay in diagnosis and therapy, or if the doctor declares they have no disease and should be discharged, it may irritate the patient and make the symptoms worse.

Meanwhile, a psychodermatology multidisciplinary team is appropriate in this condition, especially as the patient needs psychological intervention (29). We emphasize the role of a dermatology–psychiatry liaison in our treatment. The dermatological physician is to make the diagnosis and exclude organic disease, while the psychiatric physician is to manage concomitant psychiatric disease. A multidisciplinary team approach to patients with DA is essential to improve outcomes (5).

## Data Availability

The original contributions presented in the study are included in the article/Supplementary Material, further inquiries can be directed to the corresponding author/s.
